# Interferon-τ increases BoLA-I for implantation during early pregnancy in dairy cows

**DOI:** 10.18632/oncotarget.19282

**Published:** 2017-07-17

**Authors:** Zhe Zhu, Binbin Li, Yue Wu, Xiao Wang, GanZhen Deng

**Affiliations:** ^1^ College of Veterinary Medicine, Huazhong Agricultural University, Wuhan 430070, People's Republic of China

**Keywords:** interferon-τ, dairy cow, BoLA-I, nidation, early pregnancy

## Abstract

Interferon-τ (IFN-τ) signals pregnancy recognition in ruminants. We investigated the effects of IFN-τ produced by embryo trophoblastic cells (ETCs) on expression of bovine leukocyte antigen-I (BoLA-I), a bovine analogue of human MHC-I, in endometrial luminal epithelial cells (EECs) during early pregnancy in dairy cows. Expression of *IFN-τ* and BoLA-I was increased in endometrial tissues during early pregnancy. Expression of the anti-inflammatory cytokine IL-10 was increased in endometrial tissues, while expression of the pro-inflammatory cytokine IL-6 was decreased, indicating immunosuppression. Progesterone increased *IFN-τ* expression in EECs. IFN-τ increased p-STAT1 and p-STAT3 levels in EECs, but reduced TRAF3 levels. In addition, IFN-τ increased expression of BoLA-I and IL-10, but decreased expression of IL-6 in EECs. These results indicate that IFN-τ enables stable implantation in dairy cows by increasing expression of BoLA-I, and by immunosuppression mediated by increased IL-10 and decreased IL-6 expression.

## INTRODUCTION

Interferon-τ (IFN-τ), which is a cytokine characteristic for ruminants, is an anti-luteolytic protein that is secreted by trophoblast cells during early pregnancy [1]. IFN-τ has a similar structure and function as type I IFNs; bovine IFN-τ has a molecule weight of 24 kDa and binds to the same receptor as type I IFNs (*IFNAR*)[2–4]. IFN-τ has multiple functions, including *in vitro* antiviral activity in humans, ovine and bovine [4, 5]. In addition, IFN-τ has an anti-proliferative effect on cancer cells [6], and anti-inflammatory effect during bacterial infections [7–9].

IFN-τ exerts various immunomodulatory functions, but exists only for a short time in ruminants. IFN-τ is detected from 7–8 days in pregnancy, but is quickly reduced after the conceptus is implanted in the endometrial epithelial cells [7], and its specific function during early pregnancy is not clear. In early pregnancy, estradiol and progesterone also play important roles. Estradiol appears to be essential for establishing maternal receptivity to implantation in most mammals [8]. Progesterone plays a pivotal and indisputable role in the establishment and maintenance of pregnancy in mammals and its level is predictive of subsequent pregnancy loss [9]. Estradiol and progesterone can regulate the production of IFN-τ, which can affect stable implantation.

The Major Histocompatibility Complex (MHC) class I antigen has been associated with the implantation during early pregnancy. MHC-I is designated as HLA-I in humans [10], H-2-I system in mouse [11], and BoLA-I in bovine [12]. Previous studies have indicated that MHC-I has immunosuppressive effects during early embryonic development, including inhibition of cytotoxic T cells and NK cells [13], and inducing maternal-fetal tolerance [14, 15]. The maternal-fetal tolerance in dairy cows is associated with the expression of BoLA-I [16]. Based on the important regulatory function of MHC- I in reproduction [17, 18], the IFN-τ role in regulating embryo implantation needs to be elucidated.

This study aimed to analyze the expressions of IFN-τ in trophoblasts, and to investigate the IFN-τ effect on BoLA- I expression in endometrial luminal epithelial cells (EECs).

## RESULTS

### Histological changes in endometrial tissues of pregnant and non-pregnant cows

**Figure 1:**
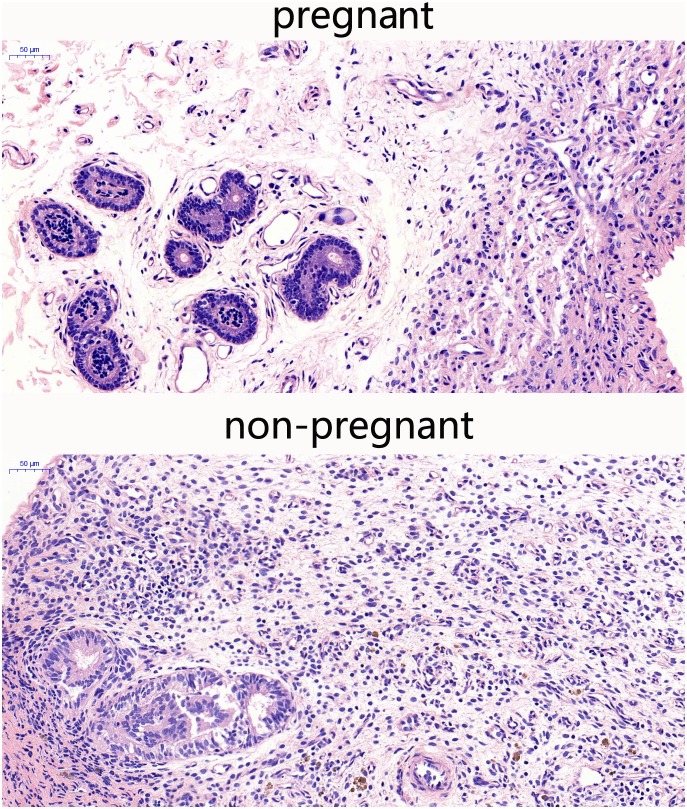
Histopathological sections of endometrial tissues (HE,×200) Endometrial tissues from pregnant and non-pregnant cows were processed for histological evaluation.

Endometrial tissues were harvested from early pregnant and non-pregnant dairy cows, sliced, and tissues slices were subjected to hematoxylin and eosin staining. As expected, endometrial tissues of early pregnant cows showed a normal structure. Early pregnant endometrial tissues showed that the luminal epithelial cells clearance was increased, and the tissue structure was loose compared with the non-pregnant cows (Figure 1), indicating that the tissue was prepared for the fetal growth. The uterus endocrine glands and the artery blood vessel were increased, preparing for compensatory nutrition for the fetal growth. Inflammatory cells, such as neutrophils and macrophages were not found, indicating a suppressed immune state in early pregnant cows.

### Production of IL-10 and IL-6 in endometrial tissues

**Figure 2:**
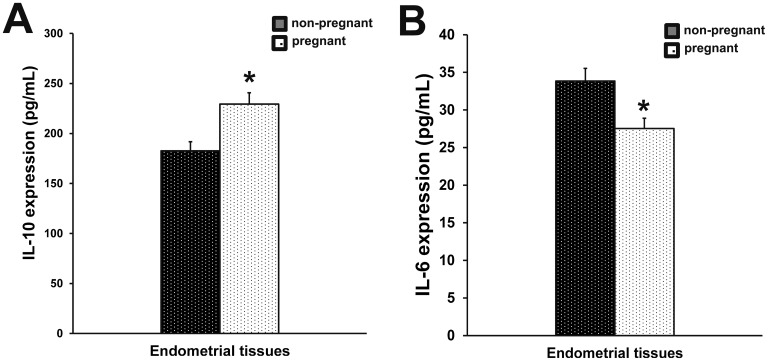
Production of IL-10 and IL-6 in endometrial tissues IL-10 **(A)** and IL-6 **(B)** expression in endometrial tissues of pregnant and non-pregnant cows was measured by ELISA. Columns represent the means. Bars represent the S.E. ^*^P<0.05 values differ significantly from the control group; ^**^P<0.01 values differ extremely significantly from the control group.

To evaluate the immune status of the uterus, IL-10 and IL-6 levels were analyzed. Compared with non-pregnant cows, the IL-6 production (P<0.05) (Figure 2B) was decreased in early pregnant cows, while the IL-10 production was elevated, indicating an immune suppression (P<0.05) (Figure 2A). However, the differences in IL-6 and IL-10 production did not reach a statistical difference.

### *IFN-τ* expression in endometrial tissues and ETCs

**Figure 3:**
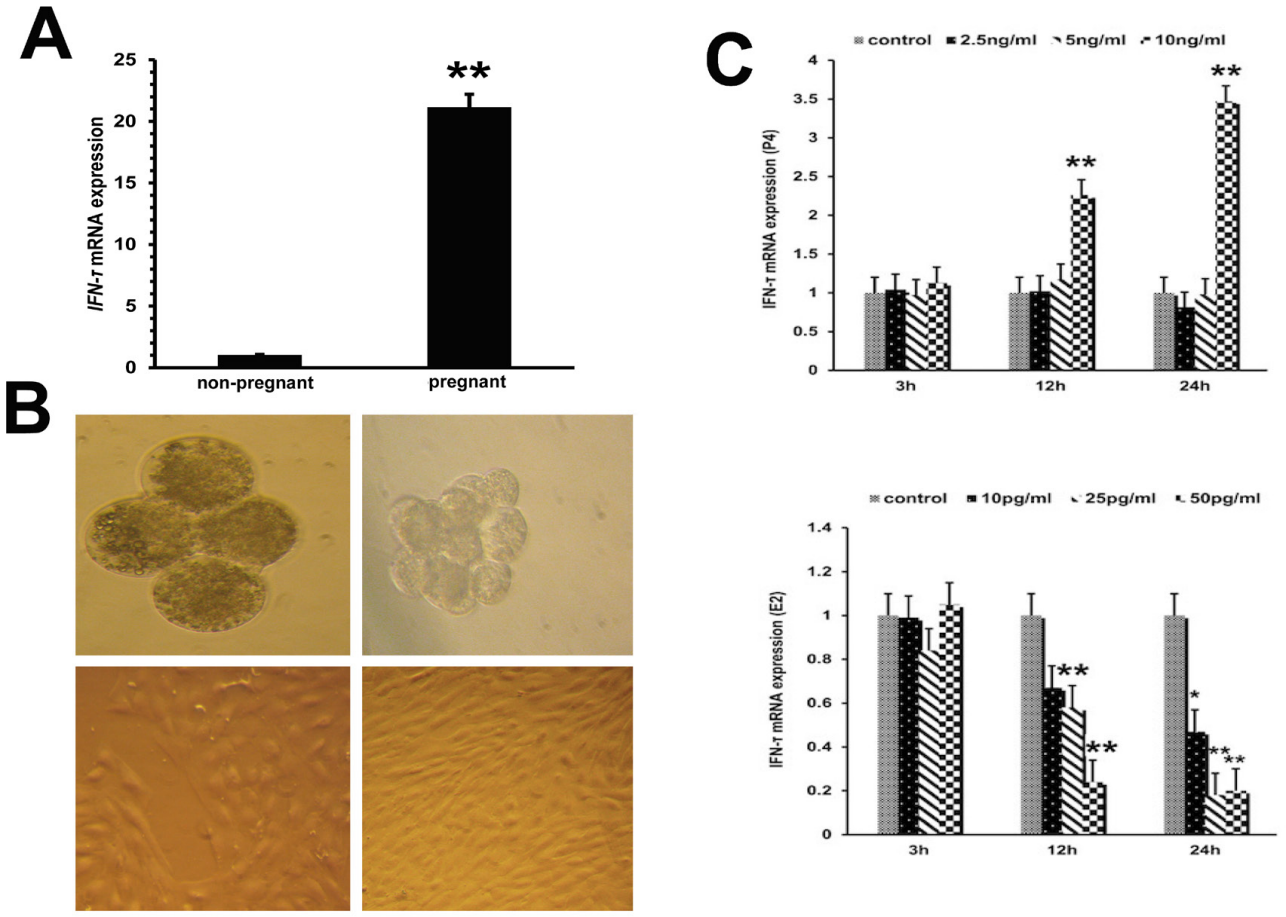
*IFN-τ* gene expression in embryo trophoblastic cells **(A)** *IFN-τ* mRNA levels in endometrial tissues of pregnant and non-pregnant cows were quantified by RT-qPCR. **(B)** Parthenogenetic embryo activation and embryo trophoblastic cell culture (40×). **(C)** Effects of progesterone and estradiol on *IFN-τ* mRNA expression in ETCs. ^*^P<0.05 values differ significantly from the control group; ^**^P<0.01 values differ extremely significantly from the control group.

As shown in Figure 3A, compared with non-pregnant dairy cows, the *IFN-τ* level was significantly increased in early pregnant cows (*P*<0.01). To investigate the mechanisms regulating the IFN-τ expression, *IFN-τ* levels were analyzed in ETCs treated with progesterone or estradiol in order to simulate endo-environment of implantation (Figure 3B). Compared with the control group, progesterone remarkably increased the mRNA level of *IFN-τ* (*P*<0.01), whereas *IFN-τ* mRNA expression was significantly reduced (P<0.01) in estradiol-treated ETCs (Figure 3C).

### BoLA-I expression in endometrial tissues

**Figure 4:**
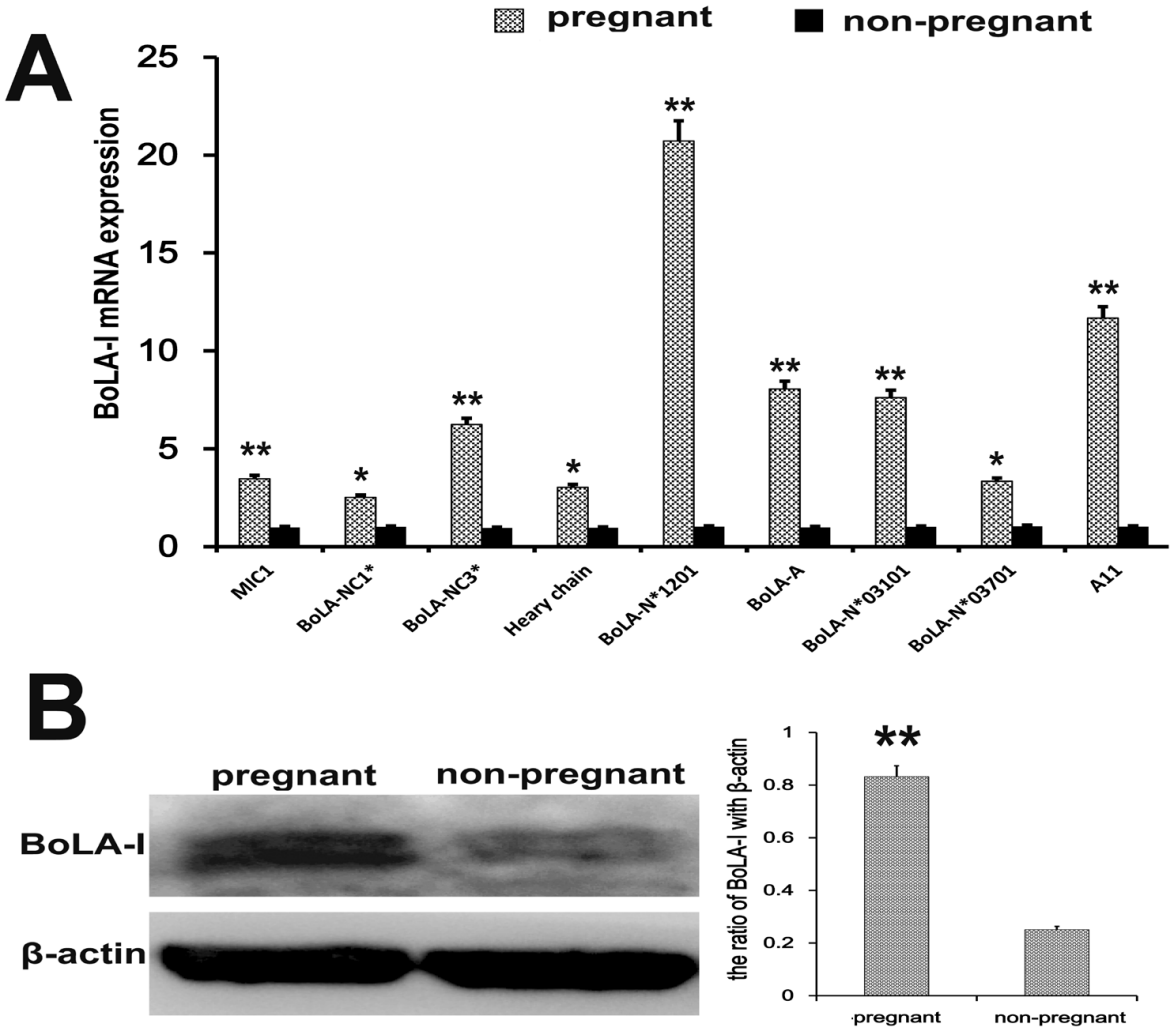
BoLA-I expression in endometrial tissues **(A)** *MIC1*, BoLA-NC1^*^, BoLA-NC3^*^, *Heavy chain*, BoLA-N^*^01201, *BoLA-A*, BoLA-N^*^03101, BoLA-N^*^03701 and *A11* mRNA levels in endometrial tissues of pregnant and non-pregnant cows were quantified by RT-qPCR. **(B)** Western blotting was performed to detect the BoLA-I protein levels in endometrial tissues of pregnant and non-pregnant cows.^*^P<0.05 values differ significantly from the control group; ^**^P<0.01 values differ extremely significantly from the control group.

Since *BoLA-I* is a highly polymorphic gene, analyzing expression of all *BoLA-I* genes is challenging, particularly because the precise number of *BoLA-I* loci remains unknown, and the *BoLA-I* genes undergo interlocking recombination. The mRNA expression of MIC1, BoLA-NC3^*^ BoLA-A, BoLA-N^*^03101, BoLA-N^*^01201 and *A11* was significantly increased in early pregnant cows (*p*<0.01) (Figure 4A). In addition, the mRNA expression of BoLA-NC1^*^, *Heavy chain*, BoLA-N^*^03701, was up-regulated (P<0.05) (Figure 4A). We also measured the protein levels of BoLA-I by western blotting. The protein expression of BoLA-I was increased in early pregnant cows (*p*<0.01) (Figure 4B).

### TRAF3, STAT1, and STAT3 expression in endometrial tissues

**Figure 5:**
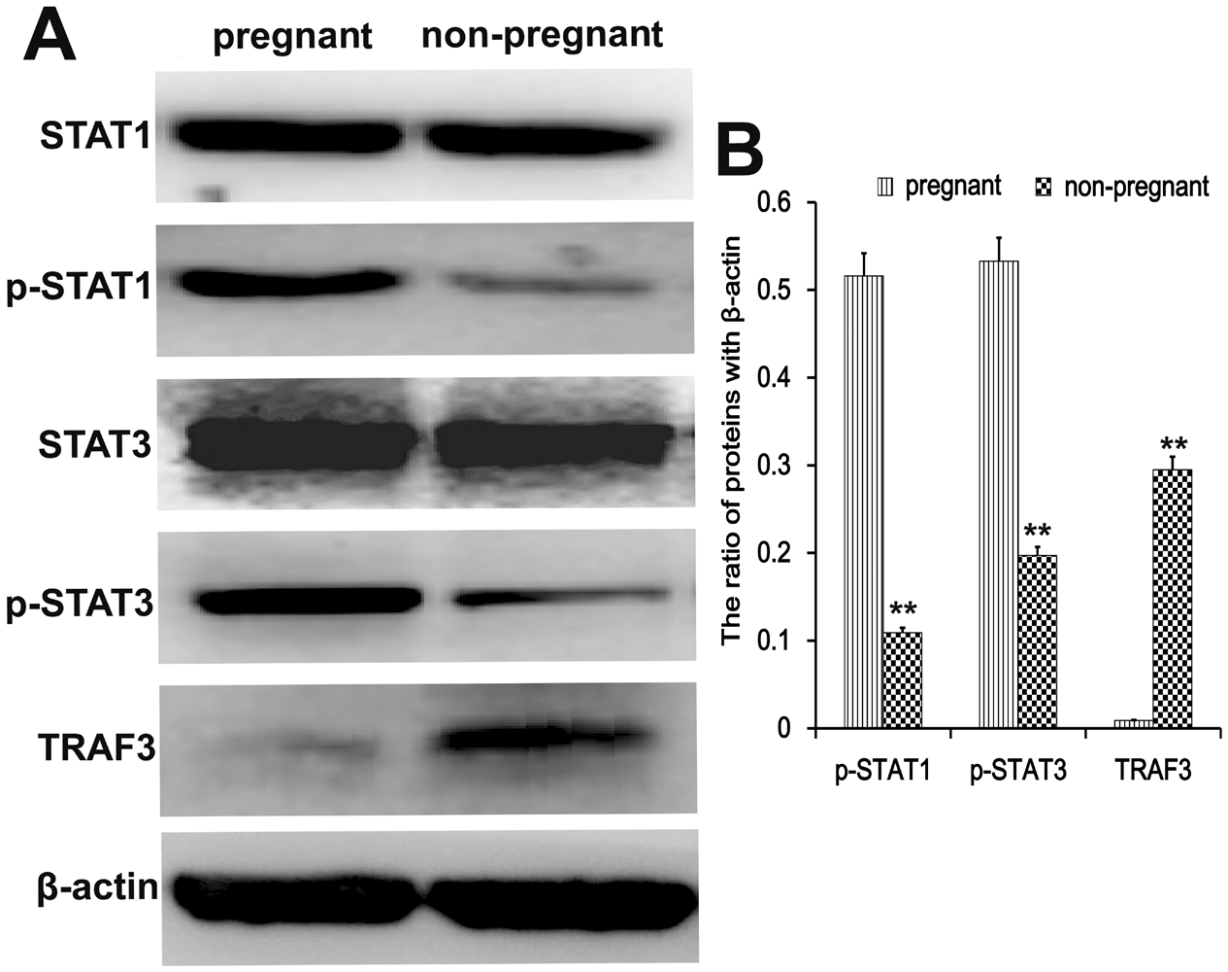
Transcription factor analysis in endometrial tissues Western blots were performed to detect the TRAF3, STAT1, and STAT3 protein levels in endometrial tissues of pregnant and non-pregnant cows. ^*^P<0.05 values differ significantly from the control group; ^**^P<0.01 values differ extremely significantly from the control group.

Transcription factors regulate the implantation during early pregnancy. We analyzed protein levels of the transcription factors TRAF3, STAT1, and STAT3 in endometrial tissues by western blotting. Compared to non-pregnant cows, the phosphorylated STAT1 and STAT3 levels were increased (p < 0.01) during early pregnancy, while the expression of TRAF3 was reduced (p < 0.01) (Figure 5).

### EECs identification

**Figure 6:**
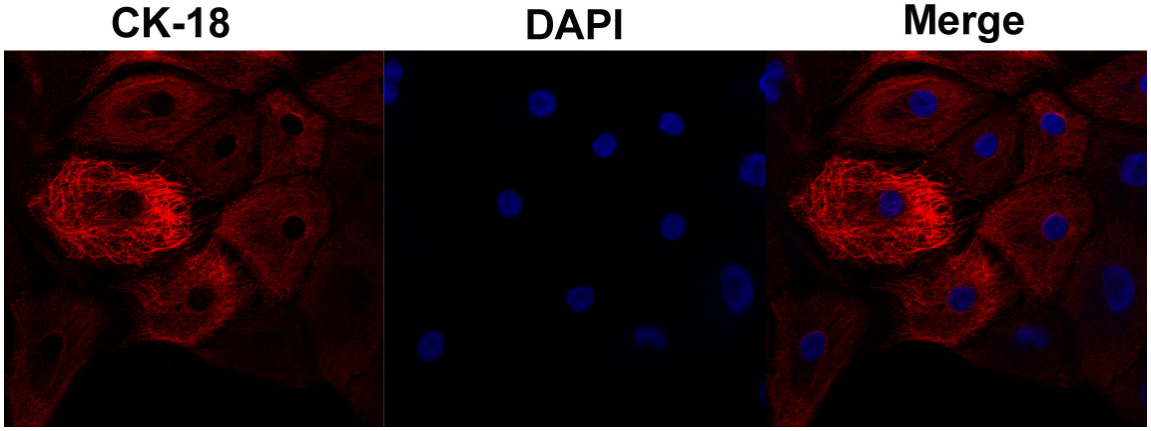
Endometrial epithelial cell culture and identification Microscopic images were captured by laser scanning confocal microscope. The EECs were verified by specific red staining with keratin CK18, which merged with nuclei that were stained blue with DAPI.

The EECs play a key role during pregnancy, and are the main target of IFN-τ. We found that EECs had an irregular round or oval shape, with large, round, but unclear nucleus and transparent cytoplasm. The EECs were verified by specific red staining with keratin CK18, and showed that the cytoplasm was clear and uniformly stained. Nuclei were stained blue with DAPI. Microscopic images were captured by LSCM and no other cell types were observed (Figure 6).

### Effect of IFN-τ on IL-10 and IL-6 production in EECs

**Figure 7:**
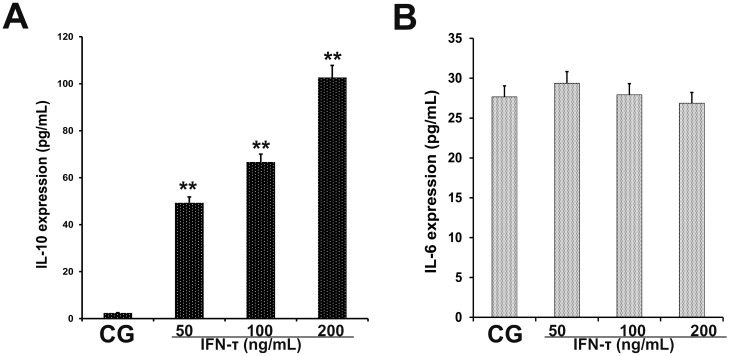
Effect of IFN-τ on IL-10 and IL-6 production in EECs ELISA was performed to detect **(A)** IL-10 and **(B)** IL-6 expression in EECs treated with control DMSO (CG) or IFN-τ. ^*^P<0.05 values differ significantly from the control group, ^**^P<0.01 values differ extremely significantly from the control group.

The EECs can produce IL-10 and IL-6 in endometrial tissues. IL-10 and IL-6 production in EECs was measured by ELISA (Figure 7). The effect was similar as in the uterus tissues. The IL-6 levels were significantly reduced in IFN-τ treated cells (p < 0.01). The IL-10 was slightly elevated in EECs treated by IFN-τ, but no statistical significance was found (p > 0.05) (Figure 7).

### IFN-τ increases BoLA-I expression in EECs

**Figure 8:**
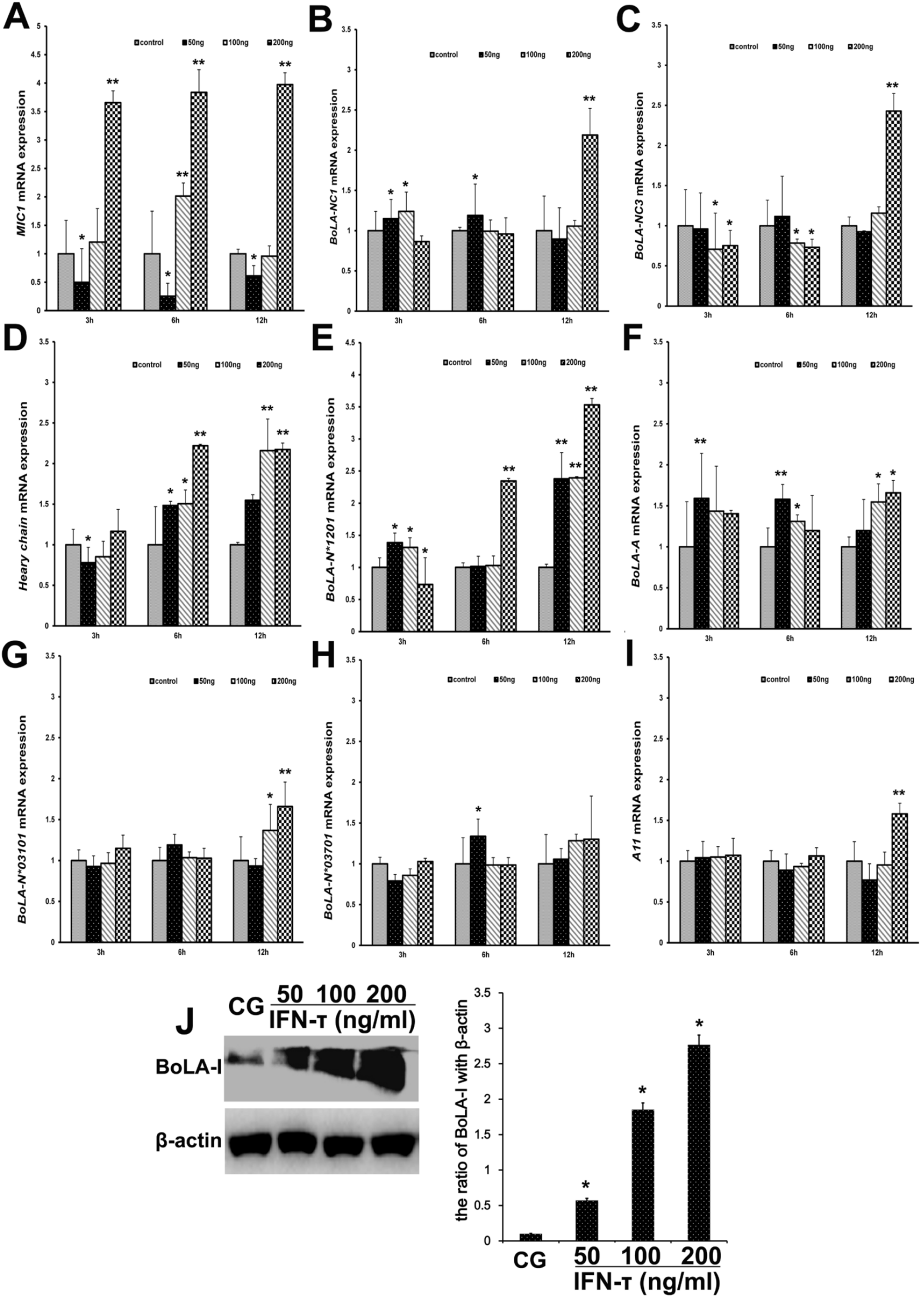
Effect of IFN-τ on BoLA-I expression in EECs **(A)** *MIC1*, **(B)** BoLA-NC1^*^, **(C)** BoLA-NC3^*^, **(D)** *Heavy chain*, **(E)** BoLA-N^*^01201, **(F)** *BoLA-A*, **(G)** BoLA-N^*^03101, **(H)** BoLA-N^*^03701 and **(I)** *A11* mRNA levels in EECs were quantified by RT-qPCR after IFN-τ treatment. **(J)** Western blots were performed to measure the BoLA-I protein levels in the control group (CG) and IFN-τ group in EECs. ^*^P<0.05 values differ significantly from the control group, ^**^P<0.01 values differ extremely significantly from the control group.

BoLA-I is expressed on EECs, which are the main target of IFN-τ. The mRNA expression of *MIC1* (Figure 8A) (*P*<0.01), BoLA-NC3^*^ (Figure 8C) (*P*<0.01) and BoLA-NC1^*^ (Figure 8B) (P<0.05) were significantly up-regulated by IFN-τ. The mRNA expression of *BoLA-A* (Figure 8D) and BoLA-N^*^01201 (Figure 8E) was significantly up-regulated by IFN-τ (*P*<0.01). Thus, the effect of IFN-τ on *BoLA-A* (Figure 8F), BoLA-N^*^03101 (Figure 8G), BoLA-N^*^03701 (Figure 8H), and *A11* (Figure 8I) mRNA expression was similar (*P*>0.05). To confirm the effect of IFN-τ on BoLA-I expression in EECs, we measured protein levels of BoLA-I by western blotting. The protein levels of BoLA-I were increased by IFN-τ in a dose-dependent manner (P<0.05) (Figure 8J).

### Effect of IFN-τ on TRAF3, STAT1, and STAT3 expression in EECs

**Figure 9:**
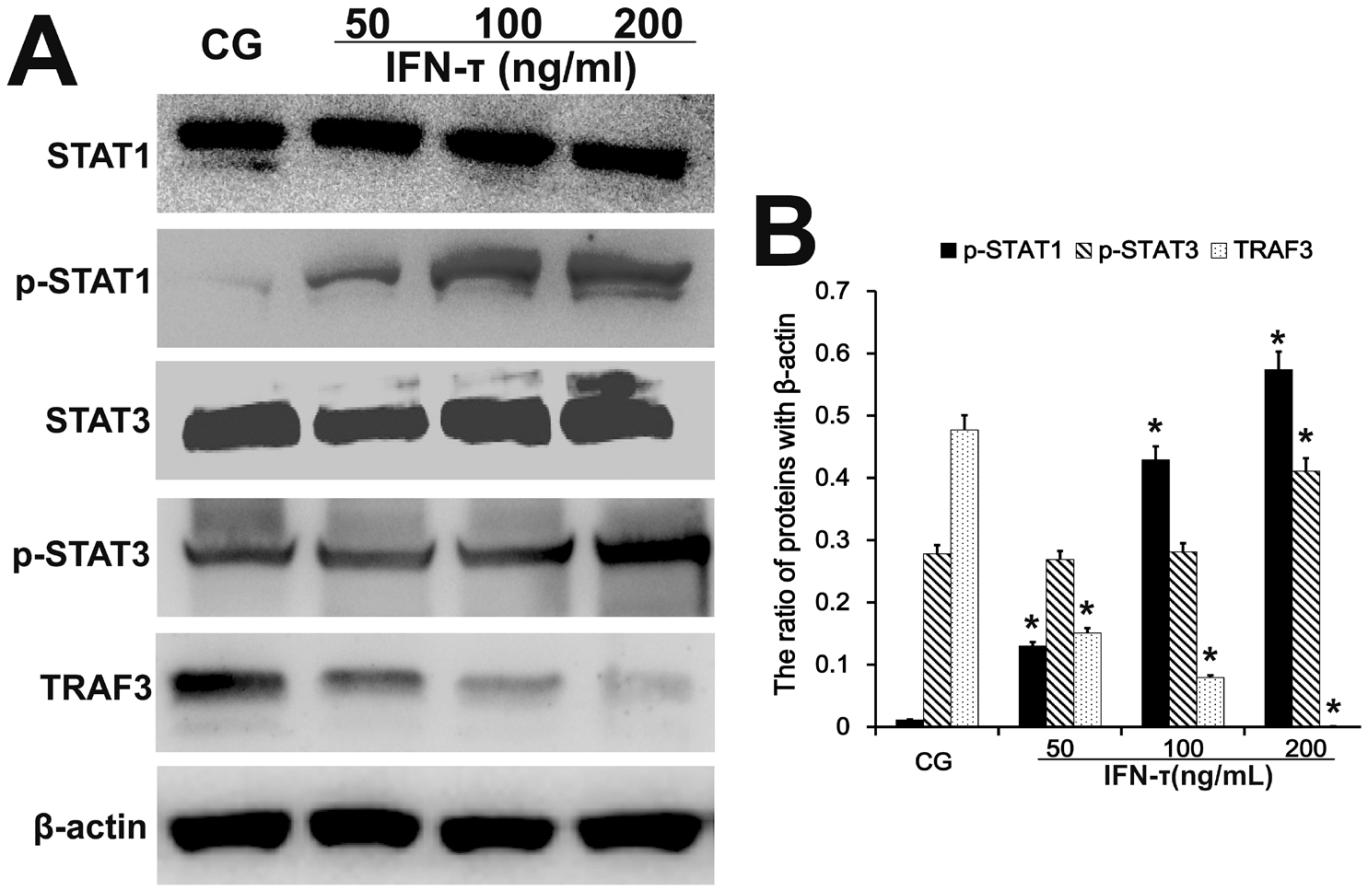
Effect of IFN-τ on transcription factor expression in EECs Western blots were performed to detect the TRAF3, STAT1 and STAT3 protein levels and their phosphorylation in the control group (CG) and IFN-τ group in EECs. ^*^P<0.05 values differ significantly from the control group, ^**^P<0.01 values differ extremely significantly from the control group.

To further analyze the effect of IFN-τ on BoLA-I expression in EECs, the relevant transcription factors TRAF3, STAT1 and STAT3 were analyzed by western blot. The protein levels of phosphorylated STAT1 and STAT3 were increased (p < 0.05) in IFN-τ treated cells, and the effect was dose dependent (Figure 9), similarly as in the uterus tissues. In contrast, the expression of TRAF3 was reduced (p < 0.05) by IFN-τ treatment in a dose-dependent manner (Figure 9).

## DISCUSSION

The implantation is an important event in the fetus formation during early pregnancy. Our results indicate that cell clearance is increased in endometrial tissues of pregnant cows to facilitate the fetal growth. The uterus endocrine glands and artery blood vessel are increased to enable fetal nutrition. In addition, immune modulation in the uterus plays a crucial role during early pregnancy. IL-6 is involved in immune defense in the uterus [19, 20], and IL-10 plays a major role in anti-immunological rejection [21, 22]. Our results showed that there were no appreciable differences in IL-10 and IL-6 production between early pregnant and non-pregnant cows, suggesting that the fetus was not rejected in the uterus by the immune system. Our previous studies have shown that IFN-τ can reduce the inflammatory cytokine expression [23, 24]. IFN-τ has been associated with the uterine immune regulation in early pregnant cows [25]. Suggesting that it may be the key factor in developing the low immune defense for stable implantation during early pregnancy.

The proper secretion of progesterone and estradiol is vital for the establishment and maintenance of pregnancy [9]. The receptors for progesterone and estradiol are expressed on ETCs, which also express IFN-τ [26–28]. IFN-τ secretion is unique to ruminant animals during early pregnancy and might be regulated by several endometrium genes (IFN-τ stimulated genes, ISGs), the expression of which is also enhanced by IFN-τ [29]. Since the specific concentrations of progesterone and estradiol regulating ETCs are not known, we used several different concentrations of estradiol and progesterone to investigate the ETCs functions. We found that ETCs exposed to a high concentration of progesterone for 12 and 24 h showed a significant increase in *IFN-τ* mRNA expression, while estradiol had the opposite effect. This result was in agreement with a previous study in sheep [30].

IFN-τ up-regulates the expression of MHC-I [31]. A previous study has demonstrated both classical and non-classical BoLA-I mRNA expression in bovine oocytes and developing pre-implantation embryos, suggesting that the expression is both gene- and stage-of-development specific [32]. The receptor of IFN-τ is located on ovine EECs [31], and BoLA-I is expressed on EECs [12]. Our study is the first to report that IFN-τ increases mRNA expression of non-classical MIC1, BoLA-NC1^*^, BoLA-NC3^*^ and classical Heavy chain and BoLA-N^*^0120 and protein levels of BoLA-I in EECs. In addition, the results in IFN-τ treated EECs are similar to the results in endometrial tissues of early pregnancy.

IFN-τ is the main messenger of communication between the ruminant female parent and the embryo, and this communication inhibits the maternal immune responses against the embryo, by adjusting cytokines and other immunomodulatory molecules [25]. Indeed, the BoLA-I promoter is responsive to IFN-τ, IFN-G, and IL4, suggesting possible roles for these cytokines in bovine pre-implantation embryo survival and/or maternal-fetal tolerance [33]. The MHC-I expression regulates the reproductive immunology in mice [11]. Our results show that IFN-τ increases BoLA-NC1^*^ expression in bovine endometrial cells; these results are supported by previous studies indicating that blastocyst *MHC-I* expression is a key component contributing to the establishment of pregnancy in cattle [34]. The endometrial immune profile of the estrous cycle favors a Th2 environment in anticipation of pregnancy, and the presence of an embryo acts to fine-tune this environment [35].

Our previous study has shown that IFN-τ inhibits inflammation by suppressing the activation of NF-κB and MAPKs [23, 24]. TRAF3 belongs to NF-κB non-canonical pathway involved in the regulation of type I IFN production [36]. However, we found the protein levels of TRAF3 in endometrial tissue of early pregnancy were reduced compared with non-pregnant cows. In addition, our results indicate that IFN-τ inhibits the TRAF3 expression in EECs, and during early pregnancy. IFN-τ might provide an alternative to other type I IFNs in the treatment of human diseases, with fewer side effects and lower cytotoxicity [37].

Transporter associated with Ag processing 1 (TAP1) and low molecular mass polypeptide 2 (LMP2) are regulated by STAT1, are essential for MHC-I function, and share a common bidirectional promoter [38]. We found that IFN-τ increases the BoLA-I and phospho-STAT1/STAT3 levels in EECs. It has been shown that MIC1 belonging to the non-classical BoLA-I family binds to the receptor NKG2D on the surface of NK cells, thus stimulating the lethal effect of NK cells [39]. A previous study has shown that STAT3 ablation in tumor cells modulates NKG2D-mediated NK cell activation, and that STAT3 directly interacts with MIC1 promoter to repress MIC1 transcription [40]. The biological effects of type I IFNs are regulated by the signaling between STAT1 and STAT3 and their relative abundance [41].

In conclusion, our study demonstrates that progesterone increases the *IFN-τ* expression in ETCs of dairy cows, while estradiol inhibits it. In addition, IFN-τ increases BoLA-I expression in EECs. IFN-τ also increases IL-10, p-STAT1 and p-STAT3 levels, but decreases IL-6 and TRAF3 levels in EECs. These results also found in early pregnant endometrial tissues indicate that IFN-τ increases BoLA-I for implantation during early pregnancy in dairy cows.

## MATERIALS AND METHODS

### Reagents

Recombinant Ovine Interferon-τ (rOvIFN-τ) was purchased from Creative Bioarray (NY, USA). Fetal bovine serum (FBS) was purchased from SAFC Biosciences (Brooklyn, AUS). Bovine IL-10 and IL-6 enzyme-linked immunosorbent assay (ELISA) kits were obtained from Biolegend (San Diego, CA, USA). Rabbit TRAF3, STAT1, and STAT3 antibodies, and monoclonal antibodies against p-STAT1 and p-STAT3 were purchased from Cell Signaling Technology Inc (Beverly, MA, USA). HLA class I (ab23755, reacts with cow BoLA-I), and horseradish peroxidase conjugated goat anti-rabbit antibodies were provided by ABCAM Biotech Co., Ltd (Cambridge, UK). SYBR green Plus regent kit was purchased from Roche Applied Science (Mannheim, Germany). All other chemicals were of reagent grade.

### Animal studies

The dairy cows were obtained from Animal Experiment Center of Huazhong Agricultural University (Wuhan, China). All experimental procedures involving animals and their care conformed to the Guide for the Care and Use of Laboratory Animals of National Veterinary Research. This study was approved by the Huazhong Agricultural University Animal Care and Use Committee. The dairy cows were anesthetized with sodium pentobarbital to minimize suffering. The ovaries, endometrial epithelium (for cell culture) and endometrial tissues of non-pregnant cows were collected before ovulation. Endometrial tissues of early pregnant cows were collected during the implantation (Day9 to Day25, n = 3). Pregnancy was induced by artificial insemination (AI); the day of insemination was designated as Day 0 of pregnancy [42, 43].

### Embryo trophoblastic cells (ETCs) culture and treatment

The procedures for oocyte acquisition and maturation were reported by Presicce and Yang [44]. For the maturation culture, cumulus-enclosed oocytes were cultured in 100 μL droplets of maturation medium (20–25 oocytes per droplet) covered with mineral oil at 37°C in 5% CO_2_ humidified air. The maturation medium consisted of bicarbonate medium M-199 with Earle's salts, 25 mM HEPES, and 7.5% FBS, supplemented with 0.5 μg/mL ovine follicle-stimulating hormone, 5.0 μg/mL ovine luteinizing hormone, 1.0 μg/mL estradiol, and 0.25 mM pyruvate. The methods described by Susko-Parrish were employed for parthenogenetic activation [45]. The oocytes were activated by a 5 min exposure to Ca-ionophore A23187 at room temperature followed by culture in 2.5 mM 6-dimethylaminopurin, for 3.5 h [46]. After washing, the treated oocytes were cultured in KSOM with 0.1% BSA for an additional 48 h. The culture environment consisted of 5% CO_2_ humidified air, or 5% O_2_, 5% CO_2_ and 90% N_2_ at 37°C. All cleaved embryos were further cultured for 8 days in SOF-BE1 medium, which consisted of 20% FBS. The primary trophectoderm cultures were started after removing the zona pellucida of the 8-day-old parthenogenetic blastocysts with 25-gauge hypodermic needles; the punctured blastocysts helped the ETCs adhere to the wall of the culture dish. Healthy ETCs emerged from the central collapsed blastocyst mass after 2 days and grew out rapidly as a tightly knit epithelial monolayer. The ETCs were treated with progesterone (2.5 ng/mL, 5 ng/mL and 10 ng/mL) or estradiol (10 pg/mL, 25 pg/mL, and 50 pg/mL)[47]. The control group was treated with the same amount of DMSO. All experiments were performed three times, and analyzed in triplicates.

### Endometrial luminal epithelial cells (EECs) culture and treatment

EECs were cultured as described previously [47]. The caruncular endometrial epithelium was mixed with sufficient 1% collagenase I, cut into 1-3 mm pieces, and digested for 1 h in a sealed container in a thermostatic shaker at 37.5°C and 88 rpm. After neutralization of collagenase I with FBS, the tissue pieces were placed on the bottom of a culture dish at 0.5 - 1 cm intervals. The dish was inverted, and the samples were incubated in 5% CO_2_ at 37°C for 3 h. The EECs were cultured in DMEM/F12 supplemented with 15% FBS, 2 mM L-glutamine, 50 U/mL of penicillin and streptomycin, 100 U/mL of gentamicin, and 10 ng/mL EGF, and maintained in a 5% CO_2_ humidified incubator at 37°C. The EECs were identified by keratin 18 with Laser Scanning Confocal Microscope. The EECs were treated with 50 ng/mL, 100 ng/mL or 200 ng/mL IFN-τ [48]. The control group was treated with the same amount of DMSO. All experiments were performed three times, and analyzed in triplicates.

### Quantitative real-time PCR

Total RNA was isolated by the TRIZOL Reagent, and converted into cDNA with PrimeScript 1ST Strand cDNA Synthesis Kit according to the manufacturer's instructions (Takara, Japan). The Primer Premier software (PREMIER Biosoft International, USA) was used to design specific primers (Table 1). Quantitative real-time PCR was performed on StepOne real-time PCR System (Life Technologies Corp) with the FastStart Universal SYBR Green Master (Roche, Germany) in a 25-μl reaction. Each sample was measured in triplicates. Results (fold changes) were expressed as 2^-ΔΔCt^. β-actin was used as a reference gene.

**Table 1:** Primers for RT-qPCR

Gene	Primer	Objective segment size	GenBank accession no.
β-actin	F 5’-TGGACTTCGAGCAGGAGAT-3’	194 bp	NM-173979.3
	F 5’-CGTCACACTTCATGATGGAA-3’		
*IFN-τ*	F 5’-TGAACAGACTCTCTCCTCATCCC-3’	151 bp	NM-001015511
	R 5’-TGGTTGATGAAGAGAGGGCTCT-3’		
*BoLA-A*	F 5ʹ-GGAGACGCAGAGAACTAAGGA-3ʹ	194 bp	NM_001040554
	R 5ʹ-TCGTTCAGGGCGATGTAA-3ʹ		
*Heavy chain*	F 5ʹ-TATGTGGACGACACGCAGT-3ʹ	187 bp	NM_001193296
	R 5ʹ-TCGCTCTGGTTGTAGTAGCC-3ʹ		
BoLA-NC1^*^	F 5ʹ-AGTATTGGGATCAAGAGACGC-3ʹ	181 bp	NM_001105616
	R 5ʹ-ATAGGCGTGCTGATTATACCC-3ʹ		
*MIC1*	F 5ʹ-AGAAAGGAGGCTTACATTCCC-3ʹ	199 bp	NM_001127317
	R 5ʹ-GCCTGGTAATGCTTGCTTAAC-3ʹ		
BoLA-N^*^01201	F 5ʹ-GGAGACGCGAAACTTCAAG-3ʹ	197 bp	DQ304655
	R 5ʹ-TCGTTCAGGGCGATGTAA-3ʹ		
BoLA-NC3^*^	F 5ʹ-AGATGACACGAGATGCCAAG-3ʹ	198 bp	DQ140378
	R 5ʹ-TCGTTCAGGGCGATGTAA-3		
BoLA-N^*^03101	F 5ʹ-GATGACGAGACGCGAATCT-3ʹ	193 bp	DQ140365
	R 5ʹ-GCGATGTAATCTCTGCCGT-3ʹ		
BoLA-N^*^03701	F 5ʹ-GTATTGGGATCGGAACACG-3ʹ	171 bp	DQ190938
	R 5ʹ-AGGTAATCTCTGCCGTCGTAG-3ʹ		
*A11*	F 5ʹ-GAGTATTGGGATGAGGAAACG-3ʹ	199 bp	NM_001143743
	R 5ʹ-AGGTAATCTCTGCCGTCGTAG-3ʹ		

### Western blot analysis

The total protein was extracted according to the manufacturer's recommended protocol (Vazyme, USA). The protein concentration was determined using the BCA Protein Assay Kit (Vazyme, USA). Samples with equal amounts of protein (50 μg) were fractionated on 10% SDS-PAGE, transferred to PVDF membrane, and blocked in 5% skim milk in TBST for 1.5 h. The membrane was incubated with primary antibody (1:500 dilution) at 4°C overnight. After washing with TBST, the membrane was incubated with secondary antibody (1:1,500 dilution) for 2 h. Protein expression was detected using the ECL Plus Western Blotting Detection System (Image Quant LAS4000mini, USA). β-actin was used as a loading control.

### ELISA assay

The endometrial tissues were harvested and homogenized with phosphate buffered saline (PBS), centrifuged, and the supernatants were collected. The supernatants of the EECs with different treatments were also collected. The supernatants were assayed for IL-10 and IL-6 levels with enzyme-linked immunosorbent assay (ELISA) kits in accordance with the manufacturer's instructions (BioLegend, San Diego, CA, USA).

### Statistical analysis

The results were analyzed using GraphPad Prism 5 (GraphPad InStat Software, San Diego, CA, USA). Comparisons among the groups were performed with one-way and two-way ANOVA. Data were expressed as the mean ± standard error (SEM). *P* values <0.05 are considered to indicate a statistically significant difference.

## Abbreviations

IFN-τ: Interferon-τ
MHC-I: Major Histocompatibility Complex I
BoLA-I: bovine leukocyte antigen I
ETCs: embryo trophoblastic cells
EECs: endometrial luminal epithelial cells

## References

[1] Kimura K, Spate LD, Green MP, Murphy CN, Seidel GE, Roberts RM (2004). Sexual dimorphism in interferon-tau production by in vivo-derived bovine embryos. Mol Reprod Dev.

[2] Alexenko AP, Ealy AD, Bixby JA, Roberts RM (2000). A classification for the interferon-tau. J Interferon Cytokine Res.

[3] Brooks K, Spencer TE (2015). Biological roles of interferon tau (IFNT) and type I IFN receptors in elongation of the ovine conceptus. Biol Reprod.

[4] Pontzer CH, Yamamoto JK, Bazer FW, Ott TL, Johnson HM (1997). Potent anti-feline immunodeficiency virus and anti-human immunodeficiency virus effect of IFN-tau. J Immunol.

[5] Pontzer CH, Torres BA, Vallet JL, Bazer FW, Johnson HM (1988). Antiviral activity of the pregnancy recognition hormone ovine trophoblast protein-1. Biochem Biophys Res Commun.

[6] Pontzer CH, Bazer FW, Johnson HM (1991). Antiproliferative activity of a pregnancy recognition hormone, ovine trophoblast protein-1. Cancer Res.

[7] Imakawa K, Kim MS, Matsuda-Minehata F, Ishida S, Iizuka M, Suzuki M, Chang KT, Echternkamp SE, Christenson RK (2006). Regulation of the ovine interferon-tau gene by a blastocyst-specific transcription factor, Cdx2. Mol Reprod Dev.

[8] Gambino YP, Maymo JL, Perez Perez A, Calvo JC, Sanchez-Margalet V, Varone CL (2012). Elsevier Trophoblast Research Award lecture: molecular mechanisms underlying estrogen functions in trophoblastic cells--focus on leptin expression. Placenta.

[9] Stevenson JS, Tiffany SM, Inskeep EK (2008). Maintenance of pregnancy in dairy cattle after treatment with human chorionic gonadotropin or gonadotropin-releasing hormone. J Dairy Sci.

[10] Garboczi DN, Utz U, Ghosh P, Seth A, Kim J, VanTienhoven EA, Biddison WE, Wiley DC (1996). Assembly, specific binding, and crystallization of a human TCR-alphabeta with an antigenic Tax peptide from human T lymphotropic virus type 1 and the class I MHC molecule HLA-A2. J Immunol.

[11] Thompson RN, McMillon R, Napier A, Wekesa KS (2007). Pregnancy block by MHC class I peptides is mediated via the production of inositol 1,4,5-trisphosphate in the mouse vomeronasal organ. J Exp Biol.

[12] Townson DH (2006). Immune cell-endothelial cell interactions in the bovine corpus luteum. Integr Comp Biol.

[13] Yao GD, Shu YM, Shi SL, Peng ZF, Song WY, Jin HX, Sun YP (2014). Expression and potential roles of HLA-G in human spermatogenesis and early embryonic development. PLoS One.

[14] Loustau M, Wiendl H, Ferrone S, Carosella ED (2012). conference: the 15-year milestone update. HLA-G.

[15] Hutter H, Dohr G (1998). HLA expression on immature and mature human germ cells. J Reprod Immunol.

[16] Davies CJ, Eldridge JA, Fisher PJ, Schlafer DH (2006). Evidence for expression of both classical and non-classical major histocompatibility complex class I genes in bovine trophoblast cells. Am J Reprod Immunol.

[17] Rebmann V, Switala M, Eue I, Grosse-Wilde H (2010). Soluble HLA-G is an independent factor for the prediction of pregnancy outcome after ART: a German multi-centre study. Hum Reprod.

[18] Sun LL, Wang AM, Haines CJ, Han Y, Yao YQ (2011). Down-regulation of HLA-G attenuates cleavage rate in human triploid embryos. J Reprod Infertil.

[19] Sierra-Mondragon E, Gomez-Chavez F, Murrieta-Coxca M, Vazquez-Sanchez EA, Martinez-Torres I, Cancino-Diaz ME, Rojas-Espinosa O, Cancino-Diaz JC, Reyes-Sanchez JL, Rodriguez-Munoz R, Rodriguez-Martinez S (2015). Low expression of IL-6 and TNF-alpha correlates with the presence of the nuclear regulators of NF-kappaB, IkappaBNS and BCL-3, in the uterus of mice. Mol Immunol.

[20] Fang X, Wong S, Mitchell BF (2000). Effects of LPS and IL-6 on oxytocin receptor in non-pregnant and pregnant rat uterus. Am J Reprod Immunol.

[21] Niu J, Yue W, Song Y, Zhang Y, Qi X, Wang Z, Liu B, Shen H, Hu X (2014). Prevention of acute liver allograft rejection by IL-10-engineered mesenchymal stem cells. Clin Exp Immunol.

[22] Hirayama S, Sato M, Loisel-Meyer S, Matsuda Y, Oishi H, Guan Z, Saito T, Yeung J, Cypel M, Hwang DM, Medin JA, Liu M, Keshavjee S (2013). Lentivirus IL-10 gene therapy down-regulates IL-17 and attenuates mouse orthotopic lung allograft rejection. Am J Transplant.

[23] Wu H, Zhao G, Jiang K, Chen X, Rui G Qiu C, Guo M, Deng G (2016). IFN-tau alleviates lipopolysaccharide-induced inflammation by suppressing NF-kappaB and MAPKs pathway activation in mice. Inflammation.

[24] Zhao G, Wu H, Jiang K, Rui G Zhu Z, Qiu C, Guo M, Deng G (2016). IFN-tau inhibits S. aureus-induced inflammation by suppressing the activation of NF-kappaB and MAPKs in RAW 264.7 cells and mice with pneumonia. Int Immunopharmacol.

[25] Ott TL, Gifford CA (2010). Effects of early conceptus signals on circulating immune cells: lessons from domestic ruminants. Am J Reprod Immunol.

[26] Bazer FW, Burghardt RC, Johnson GA, Spencer TE, Wu G (2008). Interferons and progesterone for establishment and maintenance of pregnancy: interactions among novel cell signaling pathways. Reprod Biol.

[27] Ealy AD, Larson SF, Liu L, Alexenko AP, Winkelman GL, Kubisch HM, Bixby JA, Roberts RM (2001). Polymorphic forms of expressed bovine interferon-tau genes: relative transcript abundance during early placental development, promoter sequences of genes and biological activity of protein products. Endocrinology.

[28] Bukovsky A, Cekanova M, Caudle MR, Wimalasena J, Foster JS, Henley DC, Elder RF (2003). Expression and localization of estrogen receptor-alpha protein in normal and abnormal term placentae and stimulation of trophoblast differentiation by estradiol. Reprod Biol Endocrinol.

[29] Gray CA (2006). Identification of endometrial genes regulated by early pregnancy, progesterone, and interferon tau in the ovine uterus. Biol Reprod.

[30] Gray CA, Abbey CA, Beremand PD, Choi Y, Farmer JL, Adelson DL, Thomas TL, Bazer FW, Spencer TE (2006). Identification of endometrial genes regulated by early pregnancy, progesterone, and interferon tau in the ovine uterus. Biol Reprod.

[31] Rosenfeld CS, Han CS, Alexenko AP, Spencer TE, Roberts RM (2002). Expression of interferon receptor subunits, IFNAR1 and IFNAR2, in the ovine uterus. Biol Reprod.

[32] Doyle J, Ellis SA, O'Gorman GM, Aparicio Donoso IM, Lonergan P, Fair T (2009). Classical and non-classical Major Histocompatibility Complex class I gene expression in in vitro derived bovine embryos. J Reprod Immunol.

[33] O'Gorman GM, Al Naib A, Ellis SA, Mamo S, O'Doherty AM, Lonergan P, Fair T (2010). Regulation of a bovine nonclassical major histocompatibility complex class I gene promoter. Biol Reprod.

[34] Al Naib A, Mamo S, O'Gorman GM, Lonergan P, Swales A, Fair T (2011). Regulation of non-classical major histocompatability complex class I mRNA expression in bovine embryos. J Reprod Immunol.

[35] Oliveira LJ, Mansouri-Attia N, Fahey AG, Browne J, Forde N, Roche JF, Lonergan P, Fair T (2013). Characterization of the Th profile of the bovine endometrium during the oestrous cycle and early pregnancy. PLoS One.

[36] Oganesyan G, Saha SK, Guo B, He JQ, Shahangian A, Zarnegar B, Perry A, Cheng G (2006). Critical role of TRAF3 in the toll-like receptor-dependent and -independent antiviral response. Nature.

[37] Alexenko AP, Ealy AD, Roberts RM (1999). The cross-species antiviral activities of different IFN-tau subtypes on bovine, murine, and human cells: contradictory evidence for therapeutic potential. J Interferon Cytokine Res.

[38] Brucet M, Marqués L, Sebastián C, Lloberas J, Celada A (2004). Regulation of murine Tap1 and Lmp2 genes in macrophages by interferon gamma is mediated by STAT1 and IRF-1. Genes Immun.

[39] Raulet DH, Gasser S, Gowen BG, Deng W, Jung H (2013). Regulation of ligands for the NKG2D activating receptor. Annu Rev Immunol.

[40] Bedel R, Thiery-Vuillemin A, Grandclement C, Balland J, Remy-Martin JP, Kantelip B, Pallandre JR, Pivot X, Ferrand C, Tiberghien P, Borg C (2011). Novel role for STAT3 in transcriptional Rregulation of NK immune cell targeting receptor MICA on cancer cells. Cancer Res.

[41] Tanabe Y, Nishibori T, Su L, Arduini RM, Baker DP, David M (2005). Cutting edge: role of STAT1, STAT3, and STAT5 in IFN- responses in T lymphocytes. J Immunol.

[42] Mishra B, Kizaki K, Koshi K, Ushizawa K, Takahashi T, Hosoe M, Sato T, Ito A, Hashizume K (2010). Expression of extracellular matrix metalloproteinase inducer (EMMPRIN) and its related extracellular matrix degrading enzymes in the endometrium during estrous cycle and early gestation in cattle. Reprod Biol Endocrinol.

[43] Fricke PM (2002). Scanning the future--ultrasonography as a reproductive management tool for dairy cattle. J Dairy Sci.

[44] Presicce GA, Yang X (1994). Parthenogenetic development of bovine oocytes matured in vitro for 24 hr and activated by ethanol and cycloheximide. Mol Reprod Dev.

[45] Susko-Parrish JL, Leibfried-Rutledge ML, Northey DL, Schutzkus V, First NL (1994). Inhibition of protein kinases after an induced calcium transient causes transition of bovine oocytes to embryonic cycles without meiotic completion. Dev Biol.

[46] Liu L, Ju JC, Yang X (1998). Parthenogenetic development and protein patterns of newly matured bovine oocytes after chemical activation. Mol Reprod Dev.

[47] Xiao CW, Goff AK (1998). Differential effects of oestradiol and progesterone on proliferation and morphology of cultured bovine uterine epithelial and stromal cells. J Reprod Fertil.

[48] Wang B, Goff AK (2003). Interferon-tau stimulates secretion of macrophage migration inhibitory factor from bovine endometrial epithelial cells. Biol Reprod.

